# Physiotherapeutic evaluation of patients with post COVID-19 condition: current use of measuring instruments by physiotherapists working in Austria and South Tyrol

**DOI:** 10.1186/s40945-022-00147-0

**Published:** 2022-09-15

**Authors:** Claudia Spiegl, Natalia Schiefermeier-Mach, Erika Schifferegger, Claudia Wiederin, Barbara Scheiber

**Affiliations:** 1 Department of Physiotherapy, Health University of Applied Sciences Tyrol, FH Gesundheit Tirol, Innrain 98, 6020 Innsbruck, Austria; 2 Unit for Research and Innovation, Health University of Applied Sciences Tyrol, FH Gesundheit Tirol, Innrain 98, 6020 Innsbruck, Austria

**Keywords:** Post COVID-19 condition, Physical therapy specialty, Survey, Physical and rehabilitation medicine, Physiotherapeutic assessments, Measurement instrument

## Abstract

**Background:**

The implementation of standardized assessments in physiotherapeutic practice strongly supports diagnostic and treatment plans. Previous studies reported insufficient usage of standardized assessments due to lack of time, lack of knowledge, lack of resources and other barriers. Physiotherapy in outpatient settings became essential for the rehabilitation of patients with post COVID-19 condition but it remains unknown to what extent assessments are implemented into the evaluation of these patients. In this study, we explored the current use and barriers regarding the implementation of physiotherapeutic assessments to evaluate patients with post COVID-19 condition.

**Methods:**

A cross-sectional online survey was carried out among 180 physiotherapists working in outpatient settings in Austria and South Tyrol.

**Results:**

The majority of physiotherapists (88%) indicated that standardized assessments are useful, though less than a fifth of participants actually implement assessments in practice. Among implementation barriers, “insufficient experience” (41.8%) and “lack of knowledge” (36.6%) were mentioned most often. Concerning specific post COVID-19 assessments, the evaluation of “physical and respiratory function”, “quality of life” and “activities of daily living” were stated to be of particular relevance.

**Conclusions:**

Our study revealed a low implementation rate and identified the main barriers regarding the non-usage of standardized assessments for post COVID-19 patients.

**Trial registration:**

The Private University for Health Sciences and Health Technology (UMIT TIROL), and the Research Committee for Scientific Ethical Questions granted approval for the survey (RCSEQ, Hall in Tirol, Austria, Number 2834).

**Supplementary Information:**

The online version contains supplementary material available at 10.1186/s40945-022-00147-0.

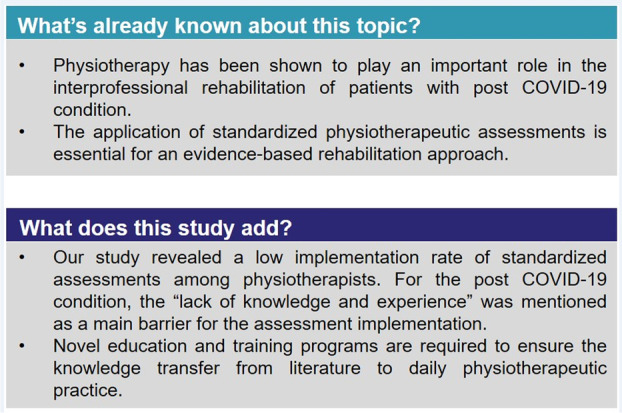



## Background

COVID-19 (coronavirus disease 2019) is documented as a multi-organ disease with a wide spectrum of acute, subacute and long-term symptoms and affects several body systems [1]. In a high number of COVID-19 survivors, persistent symptoms are reported even weeks or months after the acute infection, which are classified by the World Health Organization (WHO) as “post COVID-19 condition” [2–4]. Also, the number of people who need treatment due to post COVID-19 condition is assumed to be rising [5, 6]. Since January 2021, the ICD-10 (International Statistical Classification of Diseases and Related Health Problems 10th Revision) catalogue of the WHO includes a separate diagnosis code for post COVID-19 condition, namely U09.9 [7].

Post COVID-19 condition is associated with a broad spectrum of long-term complications. This can affect the respiratory, cardiovascular, neuromuscular, gastrointestinal, and psychological systems of the body, as well as functional abilities and participation in daily life (International Classification of Functioning, Disability and Health (ICF)) [8–11]. The most commonly reported impairment is a respiratory compromise with reduced lung function [12–20]. Long-term fatigue is also a prevalent symptom in post COVID-19 condition reported to last for about 6 months from the onset of the acute disease [2, 21]. Further symptoms of neuromuscular impairment include decreased muscle strength and reduced sensory function [10, 22, 23]. Apart from physical impairments, psychological deteriorations, limitations in activity and considerably reduced quality of life are repeatedly reported in post COVID-19 patients [8, 10, 13, 24].

Currently, health care organization for post COVID-19 cases differs among European countries. In Austria and Italy, care is organized on a regional level due to federalism and all post COVID-19 patients are advised to contact a general practitioner (GP) for a primary clinical assessment. Patients with serious, possibly life-threatening symptoms are further referred to acute services, whereas persons with no serious but more complex symptoms are referred to specialized outpatient clinics. In the case of one dominant symptom, a treatment or a self-management strategy can be directly prescribed by the GP. These patients can also be relegated to (a) a respective specialist (pulmonologist, cardiologist or other), (b) multidisciplinary specialized rehabilitation programs (c) individually practicing medical healthcare providers such as physiotherapists, occupational therapists, speech therapists or nutritional counsellors [25, 26]. Furthermore, patients may receive a prescription from their GP to consult a therapist of their trust. It is recommended that a secondary assessment of the patient’s current health status shall be performed in the outpatient setting [27].

Physiotherapy has proven to be extremely relevant for the treatment of post COVID-19 patients. Physiotherapy may help not only to restore the body functions and structures but also to regain personal activity and participation [11, 28]. Benefits of physiotherapy for post COVID-19 condition were reported as one part of multi-professional rehabilitation program in acute and post-acute inpatient settings, in specialized outpatient clinics as well as in individual physiotherapy practices [25, 29–31]. Moreover, the rehabilitation of patients suffering from multiple symptoms and functional disabilities due to post COVID-19 condition is still a new and developing field that requires a combination of novel and well-established rehabilitation approaches. The European Region of the World Confederation for Physical Therapy (ER-WP) has developed and published core standards of physiotherapy practice and recommends performing physiotherapeutic assessments with standardized measure instruments for diagnostic and prognostic purposes and evaluating changes in the patients’ health status [27]. The development and use of standardized secondary assessments is an essential part of the evidence-based practice (EBP). They are used as a tool to ensure a transparent physiotherapeutic decision-making process, for diagnostic purposes and can help to give physiotherapists and patients a reliable sense of prognosis. The use of assessments in clinical practice is also relevant for the professional exchange between colleagues, other health professionals, patients as well as medical insurance companies [32–34]. However, previous studies in Austria [35], Italy [36, 37] and other countries such as Canada [34, 38], the Netherlands [32], Saudi Arabia [39] and the U.S. [40] identified strong deficits in the application of secondary assessments with regard to the implementation of standards into physiotherapeutic practice. Reported key barriers in the usage are a lack of time, a lack of knowledge, the perception that outcome measures do not meet patient’s needs, a lack of resources and administrative support, low priority and a lack of professional consensus [32–37, 41]. The Austrian and Italian physiotherapy education has been largely restructured since 2005 in line with the Bologna Process and consequently changed from more technical vocational to a university-based academic profession [42, 43]. Thus, both countries have a rather short history of academic physiotherapy education and research activities. It was shown that EBP is not yet fully implemented in these countries [35–37, 44].

Our study was carried out in two neighboring geographical areas: Austria and the northern Italian province South Tyrol. Italy was the first country in Europe hit by COVID-19 in February 2020. Shortly after, the first cases of coronavirus infections were reported in western Austria. By the end of March 2020, more than 105,000 people had been tested positive in Italy [45] and over 10,000 in Austria [46]. Our study was carried out in the period of the “second COVID-19 wave” in Austria and Italy (December 2020- February 2021). At this time, patients of the “first wave” were recovering from the acute period of the disease. In some cases, symptoms persisted or newly appeared after both mild and severe COVID-19 infection, suggesting a post COVID-19 condition [2, 21, 47]. Since only the first interim recommendations existed [25], no physiotherapeutic standardized assessments were yet implemented into outpatient rehabilitation practice to directly evaluate patients with post COVID-19 condition. Regardless, there exist a number of known and validated measuring instruments that could adequately capture common symptoms on the one hand and functional abilities and participation on the other [6, 8–10, 48–50]. Where and how these secondary assessments are currently included in the outpatient physiotherapeutic practice remains unknown.

The objective of this study was to explore the perceived appropriateness and current use of standardized measuring instruments capable to assess potential symptoms of post COVID-19 patients by physiotherapists working in an outpatient setting originating from Austria and one adjacent Italian region (South Tyrol). Additional aims were to determine which measuring instruments are perceived to be useful for the evaluation and to detect relevant facilitators and barriers for assessment application for these patients.

## Methods

### Survey development

The study presented here has been part of a big project conducted by the Department of Physiotherapy at the Health University of Applied Sciences Tyrol between December 2020 and February 2021 [5].

To prepare a questionnaire, a systematic literature search was performed. It did not identify any existing instrument to evaluate the importance and reasons for using standardized assessments for patients with post COVID-19 condition. Therefore, we have modified published survey questions concerning the reasons for using assessments in general and modified those concerning post COVID-19 rehabilitation. For the development of this part of the questionnaire, we also conducted face-to-face, telephone and online conversations with three expert physiotherapists to gather experiences on the structure and purpose of the survey. The questionnaire was reviewed by three critical physiotherapists independent of the main sample and the research team. Based on their feedback on the content and the usability of the questionnaire, minor changes regarding the wording were made. The final version consisted of 11 questions divided into three sections. The first section covered socio-demographic data (2 questions), the second section provided information on education and current field of work (3 questions), while the last section surveyed the perceived appropriateness of standardized assessments for patients suffering from post COVID-19 condition as well as the reasons for or against using them (6 questions). The questionnaire contained open and closed answer formats and each question is presented on a separate page. The questions were not randomly ordered, but a filter was added to the fourth question in the third part, which directs participants to alternate the last question. During the survey, participants could jump back to the previous questions and change answers if necessary. Multiple votes were prevented by checking the IP address. Since the national language in Austria and South Tyrol is German, the survey was carried out online via *soscisurvey.de* (SoSci Survey GmbH, Munich, Germany) in German language. Participants' responses were anonymous. Incomplete questionnaires were considered for analysis if at least 60% of the answers were given. To present the results, the “Checklist for reporting the results of internet E-surveys” (CHERRIES) was followed [51]. The questionnaire in English is attached in Additional file 1. The completed CHERRIES  is attached in Additional file 2.

### Survey participants

The online survey was open to all registered physiotherapists in Austria and South Tyrol currently working in outpatient settings that had received an invitation to participate. Currently, the registry in both countries does not provide information on the percentage of physiotherapists working in inpatient or outpatient settings. For this reason, it was not possible to get a detailed number of potential participants [52, 53]. Individuals were furthermore eligible to participate if they met the following inclusion criteria: (a) at least holding a professional qualification in physiotherapy and (b) currently practicing in an outpatient setting, which includes freelance physiotherapeutic practices, outpatient clinics (public or private), outpatient rehabilitations and other outpatient work settings. Working solely in inpatient settings, practicing outside of Austria and South Tyrol and being a physiotherapy student were exclusion criteria for this study. It was not mandatory to be currently working with COVID-19 patients to participate in this survey.

As an invitation, a link to the online survey was provided with an email within social and private networks. Due to the lack of an official mailing list of physiotherapists in Austria and South Tyrol, potential participants were asked to distribute the invitation link to eligible physiotherapists within their professional networks. To address alumni within Austria and South Tyrol, physiotherapists were contacted via email, telephone through professional networks and specific social media groups on *Facebook*. Furthermore, *Physio Austria,* the Austrian physiotherapy professional association, announced the survey in their monthly newsletter. Four weeks after the first invitation a friendly reminder was sent out (Additional file 2**)**.

### Ethics

The Private University for Health Sciences and Health Technology (UMIT TIROL), and the Research Committee for Scientific Ethical Questions granted approval for the survey (RCSEQ, Hall in Tirol, Austria, Number 2834). All respondents participated anonymously and voluntarily. Filling out and submitting the online survey represented an informed consent of the participants, as described in the introduction of the questionnaire.

### Data analysis

Data were analyzed using *IBM SPSS* version 27.0.1 statistical software (IBM SPSS Inc., Chicago, USA). Questions with closed answer formats were descriptively analyzed based on frequencies and percentages. Evaluation of all available cases, including incomplete data sets, did not show high numbers of dropouts for any particular question. Answers to open questions were verbally transcribed. Two researchers (C.S. and B.S.) performed a simple content analysis to uncover recurring entries and built categories.

## Results

### Survey response

A total of 180 participants provided relevant data and were analyzed for this study (58.4% of first survey page completion (*n*=308), 21.9% of total survey views (*n*=822)). The answers of those participants who did not complete the full questionnaire (*n*=17), were considered for data analysis until their individual dropout if at least 60% of the questions were answered. The dropouts did not occur from any particular question (Additional file 2).

### Demographics

In total, 180 physiotherapists with a median age of 37.0 years and an interquartile range (IQR) of 29-44 participated in the online survey. The majority of the respondents were female (77.2%) with a median work experience of 11 years (IQR 5-20), ranging from less than one year to 38 years of work experience. The majority of respondents indicated that their highest level of education was a diploma in physiotherapy or a bachelor’s degree and reported to work in a freelance physiotherapeutic practice. Respondents’ educational and outpatient work-field characteristics are presented in Table 1.

**Table 1:** Participants' educational and work-field characteristics

Characteristics	n	%	Mdn	IQR
Gender	180			
Female	139	77.2		
Male	41	22.8		
Highest degree of qualification	180			
Diploma or Bachelor	132	73.3		
Master	43	23.9		
PhD	5	2.8		
Physiotherapeutic work experience in years	180		11	5-20
Current workplace ^a^	180	(225 total responses)		
Freelance physiotherapeutic practice	119	66.1		
Outpatient clinic (public hospital)	30	16.7		
Outpatient rehabilitation	22	12.2		
Outpatient clinic (private hospital)	9	5.0		
Other	45	20.0		

^a^ multiple responses possible

Abbreviations: n (sample size), % (percent), Mdn (Median), IQR (interquartile range)

### Assessment application

40% of the survey respondents (*n*=72) had already received inquiries from patients with post COVID-19 condition. The majority of participating physiotherapists (*n*=158; 87.8%) indicated a positive attitude towards the usage of assessments for these patients. Nevertheless, data showed that less than a fifth (*n*=30) actually use assessments to evaluate patients with post COVID-19 condition (see Additional file 3).

### Reasons for usage and non-usage of assessments

29 out of 30 participants further specified reasons for using assessment: 28 reported doing so “to monitor the physiotherapeutic rehabilitation progress” in their patients. Furthermore, they stated to appreciate the use of assessments “to develop specific treatment plans” (*n*=25). One physiotherapist stated “to use assessments for patient education”. Further mentioned reasons for using assessments are presented in Table 2.

**Table 2:** Participants' reasons for using assessments (percentages of participants by category, multiple answers possible)

	***n***=29	%
To monitor the physiotherapeutic rehabilitation progress	28	96.6
To develop a specific treatment plan	25	86.2
The comparability with other affected patients is appreciated	16	55.2
To provide a quick overview of the sequelae after a COVID-19 infection	16	55.2
The standardized process from diagnosis to the evaluation of physiotherapy treatment is appreciated	15	51.7
For patient education	1	3.4

*Abbreviations*: n (sample size), % (percent)

The main reasons for those who did not apply specific assessments to evaluate patients with post COVID-19 were “a lack of experience” in using assessments and “a lack of knowledge” of appropriate assessments. In the open section “other reasons for non-usage*”* the majority of the participants reported that they currently do not treat patients with post COVID-19 condition (*n*=33). Further single indications for the non-usage were: “impossible setting” (*n*=1), “discussion of the problem is sufficient” (*n*=1), “the osteopathic treatment is too individual for assessments” (*n*=1) and “patients have problems with perception” (*n*=1). Data regarding the mentioned reasons for non-usage are presented in Table 3.

**Table 3:** Participants' reasons for non-using assessments (percentages of participants by category, multiple answers possible)

	*n*=134	%
The experience in using assessments is not sufficient	56	41.8
No suitable assessments are known	49	36.6
The procedure takes too much time	13	9.8
No need to use assessments	1	0.8
Open indications	38	28.4

*Abbreviations*: *n* (sample size), % (percent)

### Relevant assessments

All study participants were asked to list assessments that they would consider potentially relevant to evaluate patients with post COVID-19 condition. In total, 125 mentions were given by 73 participants. Following the guidelines of Shah et al., we evaluated the answers and assigned them to the five categories: (a) evaluation of the physical function, (b) evaluation of the respiratory function, (c) quality of life, (d) activities of daily living and (e) cognitive function [9].

The most frequent statement which evaluates the physical function (category a) was “the six minute walking test (6MWT)” (*n*= 33), which is a sub-maximal exercise test to assess aerobic capacity and endurance. “The measurement of the maximum force” was also frequently mentioned (*n*=18). “The Timed Up and Go test (TUG)”, which estimates the probability of falls in elderly adults, was also mentioned quite frequently (*n*=8). The BORG scale, which allows individuals to subjectively rate their perceived exertion (RPE), was mentioned seven times. The need to assess respiratory function (category b) was mentioned by 33 participants. In particular, “the assessment of maximum inspiratory and expiratory force”, “the maximum oxygen uptake capacity”, “the maximum oxygen saturation” and “a spiroergometry” were listed.

From the point of view of the participating physiotherapists, various assessments are suitable for evaluating possible restrictions in the patients' quality of life (categorized as c, *n*=12), such as the SF-36 (Short Form 36 Health Survey Questionnaire), the HRQoL (Health Related Quality of Life) or the CRDQ/CRQ (Chronic Respiratory Disease Questionnaire). Six physiotherapists considered the evaluation of possible limitations in activities of daily living (category d) in patients with post COVID-19 to be useful. In this dimension, the BI (Barthel Index), which is used to measure performance in activities of daily living, and the PSFS (Patient Specific Functional Scale), which assesses functional ability to complete specific activities, were mentioned.

The Fatigue Severity Scale (FSS), which differentiates fatigue from clinical depression, and a psychological assessment were mentioned once. These two assessments can be assigned to the cognitive function category (e). The categorized mentions are summarized in Table 4. Other assessments mentioned (*n*=6) could not be assigned to a specific category.

**Table 4:** Participants' content analyzed used relevant assessments (multiple answers possible)

Assessment category	*n*=73	%
(a) Evaluation of physical function		
6MWT	33	45.2
Strength tests	18	24.7
Timed Up and Go	8	11.0
BORG Scale	7	9.6
(b) Evaluation of respiratory function	33	45.2
(c) Evaluation of quality of life	12	16.4
(d) Evaluation of activities of daily living	6	8.2
(e) Evaluation of cognitive function	2	2.7

*Abbreviations*: *n* (sample size), % (percent), *a-e* (assessment categories)

## Discussion

The multiple consequences of COVID-19 beyond the acute infection phase have a major impact not only on the affected individuals but also on health care systems. Previous studies indicate that a relatively high number (10-20%) of COVID-19 patients suffer from at least one symptom weeks to months after the first symptom onset [4, 10, 21, 31] and an increase in patients with post COVID-19 condition is expected [5, 6].

To the best of the author's knowledge, this is the first study to explore the current usage as well as the reasons for usage and non-usage of standardized measuring instruments for post COVID-19 patients by physiotherapists working in outpatient settings. A sample of 180 physiotherapists working in Austria and South Tyrol participated in this survey. Regarding gender distribution, the data of our survey corresponded to the current ratio of physiotherapists in Austria (75% female; 25% male) [54]. The vast majority of respondents considers the use of secondary assessments to evaluate the health status of patients suffering from post COVID-19 to be appropriate, but only a small number of them actually uses standardized assessments in daily practice. These data support previous studies that show a low level of assessment usage by physiotherapists in Austria and other countries [32–35, 41].

Furthermore, studies from Austria and Italy revealed a deficit in research activity and low engagement in EBP among physiotherapists [35–37, 44]. The relatively short history of academic education can be one reason for the low consideration of EBP and the implementation of assessments in daily practice [33]. Barriers to the implementation of assessments can be found on two levels: the single responsibility of the practicing physiotherapists (“lack of knowledge and competencies”) and on the organization level (“lack of time”, “no available instruments” and “an inappropriate work environment”) [32]. In our study, physiotherapists stated that they refrained from using assessments because they consider their experience in using them to be “insufficient” and they “lack knowledge” on how to select appropriate assessments. They also feared that the implementation of assessments would “take a lot of time”. Such assumptions can be identified as a further “lack of knowledge” barrier since several existing standardized tools can be applied in a timely manner.

Those therapists who already implemented assessments in their daily work indicated that they primarily use them “to monitor the treatment process” and “to develop a specific treatment plan” for their patients. Further reasons mentioned for the usage of assessments are “good comparability with other affected patients” and “possibility to get a quick overview of the sequelae after a COVID-19 infection”. Literature suggests that positive attitudes toward the use of standardized measurements and a belief in the benefits of their use are the most important factors in their adoption [27, 32, 41].

Due to the multidimensional characteristics of post COVID-19, it is a challenge for the practicing physiotherapists to adequately capture the symptoms of post COVID-19 that need to be treated. At the time the study was carried out (December 2020 – February 2021), there was already one COVID-19-specific assessment available to evaluate the long term patients’ needs after a COVID-19 infection, a clinically useful tool with good internal consistency for this patient group [55, 56]. Moreover, several existing assessments can be combined to address the rehabilitation needs and capture all possible impairments [48–50]. Another instrument for identifying patients suffering from slow or incomplete recovery is the Post-COVID-19 Functional Status (PCFS) which can also be used to track improvement over time. The PCFS is a suitable tool for assessing the impact of symptoms on the functional status of patients after a COVID-19 infection with excellent reliability and good construct validity [57–59].

In March 2021, a Spanish research team published a prospective surveillance model to facilitate the early identification and management of post COVID-19 patients [6]. This model provides easy-to-use guidance, which includes reliable assessments and measurements to ensure the necessary evaluation of the patients’ current health status, cut-off points and orientation regarding physiotherapeutic treatment. It is now a great challenge to implement those (and possibly other) assessments into daily physiotherapeutic practice.

In the course of our study, participants were asked to list all assessments that they considered potentially relevant to evaluate patients with post COVID-19 condition. Respondents named a wide range of potentially important assessments, which in sum covers recommended dimensions for a complete and specific functional assessment of patients with post COVID-19 condition [6, 48, 50]. Since the virus has a strong effect on the respiratory system, we have mentioned the respiratory rehabilitation with a specific question. This could have led to a bias in the most frequently mentioned assessments, which evaluate respiratory and functional parameters. Nevertheless, the answers given by the survey participants included a wide range of assessments and did not only refer to respiratory system. The condition of COVID-19 survivors is much more complex, therefore it is necessary to take other parameters into account [1]. Impairments regarding the quality of life and activities of daily living (ADL’s) are a huge problem in post COVID-19 patients [6]. Surprisingly, a relatively low number of participants in our survey mentioned assessments to evaluate the patients’ quality of life (*n*=12) and impairments in ADL’s (*n*=6). Although fatigue is considered one of the most problematic sequelae of COVID-19 [3] there was only one specific mention of the need to assess the severity of fatigue. The need to assess possible neurological consequences of post COVID-19, such as cognitive and mental health impairments, was rarely mentioned by the participants of our study. Physiotherapeutic measurements including the 6MWT, the Timed Up and Go, the BORG scale or the Barthel index are not time-consuming, require little or no equipment and are easy to administer in daily practice. To promote the use of assessments, adequate training, sufficient time resources and the transfer from literature into daily practice must be ensured. Our data clearly show that there is a strong need for further clarification of the clinical presentation of post COVID-19 condition. We have previously found that the willingness to obtain additional COVID-19-specific education among Austrian physiotherapists is very high [5]. The data presented here further underlines the importance of including usage of the evaluation methods in the advanced training to address post COVID-19 rehabilitation needs in Austria and South Tyrol.

The low number of participating physiotherapists is a limitation of this study and makes it difficult to generalize the results obtained. Furthermore, due to survey dissemination strategies, the exact number of physiotherapists working in an outpatient setting remains unclear and no response rate can be calculated. Newly implemented professional registers in Austria (approx. 17.100 registered physiotherapists) and Italy (approx. 700 physiotherapists listed in South Tyrol) do not provide information about the employment status or place of work. Therefore, the number of physiotherapists working in outpatient setting remains unknown. Another limitation is that the survey was accessible only online, which may exclude physiotherapists with lower technical affinity. In terms of gender, age and work specialization, the sample of respondents was similar to previous studies in Austria [60, 61]. Due to the online setting, the study limitations also include the absence of a researcher. Potential misunderstandings regarding specific survey items could not be identified or clarified directly with a person present. Even though a limitation within *SoSci Survey* was added, it cannot be ruled out that a person took part in the survey several times, by using different devices.

Additionally, participating physiotherapists with a personal interest in COVID-19 might have been more likely to participate in the survey and might have had more expertise than non-respondents. Yet, this is the first study capturing the current usage of standardized assessments for evaluating patients with post COVID-19 condition.

## Conclusion

In conclusion, our study shows that even though physiotherapists in Austria and South Tyrol consider standardized physiotherapeutic assessments useful for their daily practice, less than a fifth of participants routinely implement them. The most important barriers for the non-usage of assessments are “lack of experience” and “lack of knowledge”. The post COVID-19 condition is relatively new and there is a lack of experience for the best rehabilitation strategy, consequently evidence-based physiotherapy requires keeping oneself informed about newly published guidelines. Therefore, novel education and training programs are urgently required to ensure the knowledge transfer from literature to daily practice to perform standardized assessments of patients with post COVID-19 condition.

## Supplementary Information

**Additional file 1.**

**Additional file 2.**

**Additional file 3.**

## Data Availability

On request

## Abbreviations

COVID-19: coronavirus disease 2019
WHO: World Health Organization
ICD-10: International Statistical Classification of Diseases and Related Health Problems 10th Revision
ICF: International Classification of Functioning, Disability and Health
GP: general practitioner
ER-WP: European Region of the World Confederation for Physical Therapy
EBP: evidence-based practice
CHERRIES: Checklist for reporting the results of internet E-surveys
RCSEQ: Research Committee for Scientific Ethical Questions
IQR: interquartile range
n: sample size
%: percent
Mdn: Median
6MWT: Six Minute Walking Test
TUG: Timed Up and Go test
RPE: rating of perceived exertion
SF-36: Short Form 36 Health Survey Questionnaire
HRQoL: Health Related Quality of Life
CRDQ/CRQ: Chronic Respiratory Disease Questionnaire
BI: Barthel Index
PSFS: Patient Specific Functional Scale
FSS: Fatigue Severity Scale
ADL’s: activities of daily living
