# Supplementary material for: Physiotherapeutic evaluation of patients with post COVID-19 condition: current use of measuring instruments by physiotherapists working in Austria and South Tyrol
Source: Arch Physiother. 2022 Sep 15;12:21. doi: 10.1186/s40945-022-00147-0 (PMC9473730; doi:10.1186/s40945-022-00147-0)
Supplement: Supplementary file 1 — Additional file 1. [file 40945_2022_147_MOESM1_ESM.pdf]

Dear physiotherapists, dear colleagues,

the COVID-19 pandemic poses great challenges to all of us in our daily work as physiotherapists. The aim of this research project is to assess the feasibility of effective rehabilitation for patients with sequelae of COVID-19 infection in an outpatient physiotherapy setting.

We would like to ask for your help by sharing your opinion, experience and perception by filling in the questionnaire we have prepared.

The completion of this questionnaire will take 5-10 min.

All data will be collected anonymously, cannot be attributed to any individual and will be used exclusively for this research project.

By submitting the completed questionnaire, the participant agrees to take part in the study. A subsequent withdrawal of the declaration of consent is not possible due to the anonymous survey procedure.

Thank you very much for your willingness to complete this questionnaire!

FH Gesundheit Tirol/Health University of Applied Sciences Tyrol
Innrain 98
A-6020 Innsbruck, Austria
Correspondence: Barbara Scheiber, BSc, MSc
barbara.scheiber@fhg-tirol.ac.at (+43 512 5322 76763)

Part 1: Demographic data

1. What is your gender?

____

2. What is your age in years?

____

Part 2: Information on your education and current field of work

1. What is your highest completed, professionally relevant education?

□ Diploma
□ Bachelor
□ Master or equivalent
□ Doctoral degree

2. How many years of professional experience do you have as a physiotherapist?

____

3. In which of the following areas do you currently work? (Multiple answers possible)

□ Outpatient clinic (public hospital)
□ Outpatient clinic (private hospital)
□ Outpatient rehabilitation
□ Independent physiotherapy practice
□ Other: ______________

Part 3: Assessment specific questions

1. Have you had any requests for respiratory rehabilitation for patients with sequelae after a COVID-19 infection?

□ Yes
□ No

2. Do you find the application of assessments appropriate?

□ Yes
□ No

3. In your opinion, which assessments could be relevant?

____

4. Do you use assessments to evaluate patients with sequelae after COVID-19 infection?

□ Yes
□ No

5. Why do you use specific assessments to evaluate patients with sequelae after COVID-19 infection?

□ To develop a specific treatment plan.
□ To monitor the physiotherapeutic rehabilitation progress.
□ The standardised process from diagnosis to the evaluation of physiotherapy treatment is appreciated.
□ The comparability with other affected patients is appreciated.
□ To provide a quick overview of the sequelae after a COVID-19 infection.
□ Other: ______________

6. Why don't you use specific assessments to evaluate patients with sequelae after COVID-19 infection?

□ The experience in using assessments is not sufficient.
□ No suitable assessments are known.
□ The procedure takes too much time.
□ No need to use assessments.
□ Other: ______________
