# Supplementary material for: Physiotherapeutic evaluation of patients with post COVID-19 condition: current use of measuring instruments by physiotherapists working in Austria and South Tyrol
Source: Arch Physiother. 2022 Sep 15;12:21. doi: 10.1186/s40945-022-00147-0 (PMC9473730; doi:10.1186/s40945-022-00147-0)
Supplement: Supplementary file 2 — Additional file 2. [file 40945_2022_147_MOESM2_ESM.pdf]

**Checklist for Reporting Results of Internet E-Surveys (CHERRIES) (51)**

| Cherries Category | Cherries Checklist Item | Our study |
|---|---|---|
| **Design** | Describe survey design | The survey design was described in the recipient's email invitation. |
| **IRB approval and informed consent process** | IRB approval | The study was approved by RCSEQ by University for x.x. (x.x. *is used to preserve anonymity for the manuscript revision*; Number 2834). |
| | Informed consent | Participants were informed about the purpose of the study, anonymity, approximate response time of the survey, the name of the investigator and affinitions of the research team. This information was also repeated on the first page of the survey. |
| | Data protection | No personal information was collected. |
| **Development and pre-testing** | Development and testing | First, we performed a systematic literature search that did not reveal any existing survey to answer our research question. Therefore, we have modified published survey questions concerning the reasons for using assessments in general and modified those for the post COVID-19 rehabilitation. We further conducted face-to-face, telephone and online conversations with three expert physiotherapists to gather experiences on the structure and purpose of the survey. The survey was prepared in German language using a standard solution for academic online surveys "SoSci Survey" (www.soscisurvey.de).<br><br>Three critical physiotherapists independent of the main sample and the research team reviewed the first draft of the online survey. Based on their feedback on the content and the usability of the questionnaire, minor changes were made.<br><br>Each question was presented on a single page. The questions were not randomized ordered, but filters were applied when necessary. During the survey, participants could go back to the previous questions and change answers if necessary. Multiple site visits were prevented by the SoSci platform so that each IP address could only be used once. |
| **Recruitment process and description of the sample having access to the questionnaire** | Open survey versus closed survey | The survey was open to each visitor of the site who had obtained a link to the survey. |

| | | |
|---|---|---|
| | Contact mode | The initial contact with physiotherapists in Austria and South Tirol was made via email, telephone and personal conversations (about n=100). Further, potential participants were contacted through professional networks via email and telephone (about n=200). Four weeks after the first invitation a friendly reminder was sent out. |
| | Advertising the survey | The survey was advertised in the closed *Physio Austria* (about 5900 members) and *Physiotherapie Österreich* (about 6300 members) Facebook groups. Physio Austria also announced the survey in their monthly newsletter (about 7000 members). These three groups have mostly the same participants. Potential participants were also contacted via email/telephone and asked to distribute the invitation link to physiotherapists within their own professional networks. Four weeks after the first invitation a friendly reminder was sent out. |
| **Survey administration** | Web/E-mail | The survey was posted on the SoSci Web. Responses clicking on the survey link were automatically captured by the SoSci platform. |
| | Context | SoSci Survey is a standard solution for academic online surveys in Austria, Italy, Germany and other countries. |
| | Mandatory/voluntary | After the first „Introduction“ page, the survey could be voluntary filled or discontinued at any time point/question. |
| | Incentives | No incentives were offered. |
| | Time/Date | Data was collected within two months (1. December ’20– 1. February ’21) |
| | Randomization of items or questionnaires | Survey items were not randomized. |
| | Adaptive questioning | No adaptive questioning was used. |
| | Number of items | One questionnaire item per page was displayed. The total amount of questions was eleven. |
| | Number of screens (pages) | Each question was presented on a single page. |
| | Completeness check | SoSci allows users to configure a completeness check based on Java Script. A non-response option was also provided to study participants. For various questions, one response or multiple response options were available. |
| | Review step | Study participants were allowed to review and to alter their responses before submission. |

| | | |
|---|---|---|
| **Response rates** | Unique site visitor | SoSci captured IP addresses to define unique site visitors (n=838). |
| | View rate (Ratio unique site visitors/unique survey visitors) | SoSci platform calculated this information (n=822). View rate = 98%. |
| | Participation rate (Ratio unique survey page visitors/agreed to participate) | SoSci platform calculated this information (n=308). Participation rate = 38%. |
| | Completion rate (Ratio agreed to participate/finished survey to at least 60%) | SoSci platform calculated this information (n=180). Completion rate = 58%. 53% finished completely, 5% (n=17) answered at least 60% of the questionnaire. The dropouts did not occur for any particular question. |
| **Preventing multiple entries from the same individual** | Cookies used | No cookies were set. |
| | IP check | The IP address was used by SoSci to allow a user to prevent duplicate entries without a defined period of time. |
| | Log file analysis | No analysis of the log file was performed. |
| | Registration | For participating, no registration was required. |
| **Analysis** | Handling of incomplete questionnaires | All surveys were analyzed, both completed and uncompleted, but only surveys completed to at least 60% of questions were used for the final data analysis.<br>Uncompleted questions were analyzed as missing. |
| | Questionnaires submitted with an atypical timestamp | No timestamp has been recorded. |
| | Statistical correction | No statistical corrections were made. |
