# Supplementary material for: Physiotherapeutic evaluation of patients with post COVID-19 condition: current use of measuring instruments by physiotherapists working in Austria and South Tyrol
Source: Arch Physiother. 2022 Sep 15;12:21. doi: 10.1186/s40945-022-00147-0 (PMC9473730; doi:10.1186/s40945-022-00147-0)
Supplement: Supplementary file 3 — Additional file 3. [file 40945_2022_147_MOESM3_ESM.pdf]

**Data for assessment application**

Do you find the application of assessments appropriate?

| | n=180 | % |
|---|---|---|
| Yes | 158 | 87.8 |
| No | 22 | 12.2 |

Participants' evaluation of the usefulness of an assessment application

**Data for assessment usage**

Do you use assessments to evaluate patients with sequelae after COVID-19 infection?

| Assessments usage | n=168 | % |
|---|---|---|
| Yes | 30 | 17.9 |
| No | 138 | 82.1 |

Participants' evaluation of the usage of assessments
